# Why is it important how the nursing profession is perceived?

**DOI:** 10.1177/17449871251347172

**Published:** 2025-09-15

**Authors:** Pernilla Garmy

**Affiliations:** Professor, Faculty of Health Sciences, Kristianstad University, Sweden

Do you need to be creative to be a nurse? Is the nursing profession based on scientific knowledge? These and similar questions are addressed in the *Scale for the Image of Nursing Profession* (SINP), a questionnaire designed to explore public knowledge about – and perceptions of – the nursing profession.

For those of us within the field, it is self-evident that nursing is grounded in science, but it is also an art – just as Florence Nightingale expressed in the 19th century: *the art and science of nursing* (Nightingale, 1859/2010). But it is not enough that we ourselves know this –it must also be visible and acknowledged in society at large.

Despite the fact that nursing education in most parts of the world requires an academic degree, often at the bachelor’s level, the image of nursing as a practical and ‘apprenticeship-based’ profession still persists. In the study by Godino et al. (2025), a psychometric evaluation of the SINP questionnaire was conducted in an Italian context. The instrument was originally developed in Turkey, but in this study, it was translated into Italian and culturally adapted through a rigorous six-step process.

Why, then, is it important how the nursing profession is perceived? Is it not enough that we within the profession know its value? Unfortunately, it is not that simple. There is a global shortage of nurses, and to successfully recruit new professionals, the profession must be seen as both relevant and appealing. A realistic and positive image of nursing is crucial to attract future students. Furthermore, public perception plays a role in negotiating for better working conditions. Many nurses are leaving the profession due to demanding work environments and relatively low pay – an issue that urgently needs to be addressed.

The professional role of nurses has not always been accurately or contemporaneously represented in the media, despite the fact that the profession has evolved significantly over time. Gil and Baker (2021) examined why this shift in representation has been so slow, and how stereotypical portrayals affect both public understanding and nurses’ own professional identity. They argued that the male-dominated media industry contributes to the persistence of outdated stereotypes, whereas social media offers a powerful platform to rapidly spread more accurate and nuanced representations of nursing.

Patricia Benner’s (2001) classic work *From Novice to Expert* also highlights the complexity of nursing. Godina et al. (2025) emphasised the importance of understanding this complexity from a systems perspective – not least to support development pathways that are not only vertical but also allow for professional deepening and expansion.

## References

[bibr1-17449871251347172] Benner PE (2001) From novice to expert: Excellence and power in clinical nursing practice. (Commemorative ed). Upper Saddle River, NJ: Prentice Hall.

[bibr2-17449871251347172] Gil J Baker C (2021) The power of mass media and feminism in the evolution of nursing’s image: A critical review of the literature and implications for nursing practice. Journal of Medical Humanities 42: 371–386. DOI: 10.1007/s10912-019-09578-6. 31713004

[bibr3-17449871251347172] Godino L Magi CE Ricco M , et al. (2025) Italian cross-cultural validation of the Scale for the Image of the Nursing Profession. Journal of Research in Nursing. Epub head of print. doi:10.1177/17449871251347157 10.1177/17449871251347157 PMC12394205 40895595

[bibr4-17449871251347172] Nightingale F (1859/2010) Florence Nightingale’s Notes on Nursing: What it is and what it is not & Notes on Nursing for the Labouring Classes. Commemorative edition. New York: Springer.

